# Hay Fever and Its Successful Treatment

**Published:** 1885-08

**Authors:** 


Hay Fever and Its Successful Treatment by superficial organic alteration of the nasal mucous membrane. An Essay read before the Philadelphia Laryngological Society, April 24, 1885. By Charles E. Sajous, M. D., Instructor of Rhinology and Laryngology in the Post Graduate and Spring Courses, Jefferson Medical College; President of the Philadelphia Laryngological Society; Fellow of the American Laryngological Association; Corresponding Member Royal Society of Belgium, and of the Medical Society of Warsaw (Russia), etc., etc. Illustrated with thirteen wood engravings. Philadelphia: F. A. Davis, Attorney, Publisher, No. 1217 Filbert street. 1885.

This little volume of 103 pages is a contribution towards illustrating the local neurotic origin of what becomes a constitutional disturbance at certain periods, and the author claims that he was the first to demonstrate the practical value of superficial organic alteration of the nasal mucous membrane as a radical remedy for the disease.

On the principle that the most precious articles are done up in small parcels, this book recommends itself to the reader, and it is issued in good style.

